# Intranasal budesonide for rhinitis during a high airborne pollution period: a randomized controlled trial

**DOI:** 10.1186/s13223-022-00686-y

**Published:** 2022-06-20

**Authors:** Yuan Zhang, Chunguang Shan, Weiwei Liu, Yaozhong Han, Guanggang Shi, Yongjian Ma, Kerstin Wagner, Xiaoyan Tian, Lili Zhang, Allan Joseph Larona, Steven Sacavage, Kathleen Franklin, Chengshuo Wang, Luo Zhang

**Affiliations:** 1 Department of Allergy, Beijing TongRen Hospital, Capital Medical University, Beijing, China; 2 Beijing Laboratory of Allergic Diseases and Beijing Key Laboratory of Nasal Diseases, Beijing Institute of Otolaryngology, Beijing, People’s Republic of China; 3 Department of Otolaryngology Head and Neck Surgery, Beijing TongRen Hospital, Capital Medical University, Beijing, China; 4 Department of Otolaryngology Head and Neck Surgery, The Second Hospital to Hebei Medical University, Shijiazhuang, Hebei China; 5 Department of Otolaryngology Head and Neck Surgery, Cangzhou Center Hospital, Cangzhou, Hebei China; 6 Department of Otolaryngology Head and Neck Surgery, The No.2 Hospital of Baoding, Baoding, Hebei China; 7 Department of Otolaryngology Head and Neck Surgery, Shandong Provincial Hospital, Jinan, Shandong China; 8 Department of Otolaryngology Head and Neck Surgery, The No.2 People’s Hospital of Weifang, Weifang, Shandong China; 9 Johnson & Johnson Consumer Inc, Fort Washington, PA USA; 10 Johnson & Johnson Consumer China Ltd, Shanghai, China; 11 Johnson & Johnson Consumer Regional Office Asia Pacific, Singapore, Singapore

**Keywords:** Airborne pollution, Air quality index, Budesonide, Intranasal corticosteroid, Intranasal spray, Non-allergic rhinitis, Perennial allergic rhinitis, Pollution, Rhinitis, Seasonal allergic rhinitis

## Abstract

**Background:**

Air pollution may induce or reinforce nasal inflammation regardless of allergy status. There is limited direct clinical evidence informing the treatment of airborne pollution-related rhinitis.

**Objective:**

To assess the effectiveness of intranasal budesonide in adults with self-reported rhinitis symptoms triggered/worsened by airborne pollution.

**Methods:**

Adults in northern China with self-reported rhinitis symptoms triggered or worsened by airborne pollution were randomized to budesonide 256 µg/day or placebo for 10 days in pollution season (October 2019 to February 2020). The primary endpoint was the mean change from baseline in 24-h reflective total nasal symptom score (rTNSS) averaged over 10 days. The secondary endpoints were subject-assessed Global Impression of Change (SGIC), mean change from baseline in individual nasal symptom severity, and mean change from baseline in individual non-nasal symptoms of cough and postnasal drip severity. One-sided *P* < 0.0125 was considered statistically significant.

**Results:**

After an interruption by COVID-19, an interim analysis showed that the study could be ended for efficacy with n = 206 participants (103/group) since the primary efficacy endpoint demonstrated significant results. The final efficacy results showed that the 10-day-averaged rTNSS change in the budesonide group was greater than with placebo (− 2.20 vs − 1.72, *P* = 0.0107). Budesonide also significantly improved 10-day-averaged itching/sneezing change (− 0.75 vs − 0.51, *P* = 0.0009). Results for SGIC and all other individual symptoms did not show significant differences between the two groups.

**Conclusions:**

Intranasal budesonide 256 µg once daily improved the total nasal symptoms and itching/sneezing over 10 days in adults with rhinitis triggered/worsened by airborne pollution.

**Supplementary Information:**

The online version contains supplementary material available at 10.1186/s13223-022-00686-y.

## Introduction

Epidemiologic data from the World Health Organization demonstrates that globally 9 out of 10 people breathe air containing high levels of pollutants, i.e. any chemical, physical or biological agent in the indoor or outdoor environment that modifies the natural characteristics of the atmosphere [1]. Air pollution poses a major threat to health [2] and has been associated with an increased prevalence of respiratory conditions, including allergic rhinitis (AR) [3–5] and non-allergic rhinitis (NAR) [6]. Clinical research suggests that airborne irritants can reinforce allergic sensitization and exacerbate allergic reactions and that existing nasal allergies can magnify reactions to nasal irritants [7]. Airborne pollutants also appear to contribute to rhinitis symptoms regardless of allergy status [8, 9].

Intranasal steroids (INS) reduce nasal mucosa inflammation and suppress polymorphonuclear and mononuclear cell recruitment, cytokine production, and late-phase nasal reactions [10, 11]. Available guidelines and consensus statements suggest that INS may be appropriate for treating rhinitis symptoms with contribution from non-allergic etiologies such as airborne irritants or pollution [12, 13]. Budesonide, a locally active second-generation corticosteroid, has been available for decades in nasal spray form to treat AR and NAR.

While treating with INS is recommended by clinicians in managing AR and NAR, there is still a lack of direct clinical evidence demonstrating the efficacy of any INS in treating airborne pollution-related rhinitis. Hence, this study was conducted to assess the efficacy of intranasal budesonide 256 µg/day in adults with self-reported rhinitis symptoms triggered or worsened by airborne pollution.

## Methods

### Study design

This parallel-group, randomized, double-blind, placebo-controlled, multicenter study was conducted at six centers located in Beijing, Hebei, and Shandong in northern China from October 2019 to February 2020. The sites were selected for areas with a history of airborne pollution reaching at least unhealthy levels for susceptible population, i.e. AQI > 100, during winter.

### Subjects

The study targeted a real-world population of rhinitis sufferers with non-allergic or mixed allergic/non-allergic etiologies.

Eligible subjects were aged 18–80 years and required to have moderate to severe rhinitis symptoms (defined by an rTNSS ≥ 5/9), self-reported to be triggered or worsened by airborne pollution in the previous year and at screening and confirmed by designated physician-investigators in each site regardless of the presence of underlying allergies. Among patients with self-reported allergies, patients who reported that their nasal symptoms would still exacerbated during air pollution when the exposure to positive allergens was relatively stable, they would be included in this study. The living environment of all subjects was basically fixed throughout the study period. Subjects also had to have outdoor exposure during a normal winter week, including ≥ 1 h on most days. Key exclusion criteria were upper respiratory infection within 2 weeks of screening visit or currently experiencing symptoms of respiratory infection, wearing of N95 masks during days with high airborne pollution, uncontrolled asthma, diagnoses of nasal and sinus pathologies (such as nasal polyps, deviated septum, rhinosinusitis, etc.), or concurrent use of any medication to treat rhinitis, including traditional Chinese medicine or herbal therapies.

### Randomization and blinding

After screening, the eligible subjects were randomized 1:1 to budesonide or placebo. The subjects were randomized using a central randomization system prepared and managed by a third-party statistician. The investigators at each site entered the subjects into the system after obtaining consent. The patients and investigators were blind to treatment assignment. All spray containers had the same appearance.

### Intervention

**Fig. 1:**
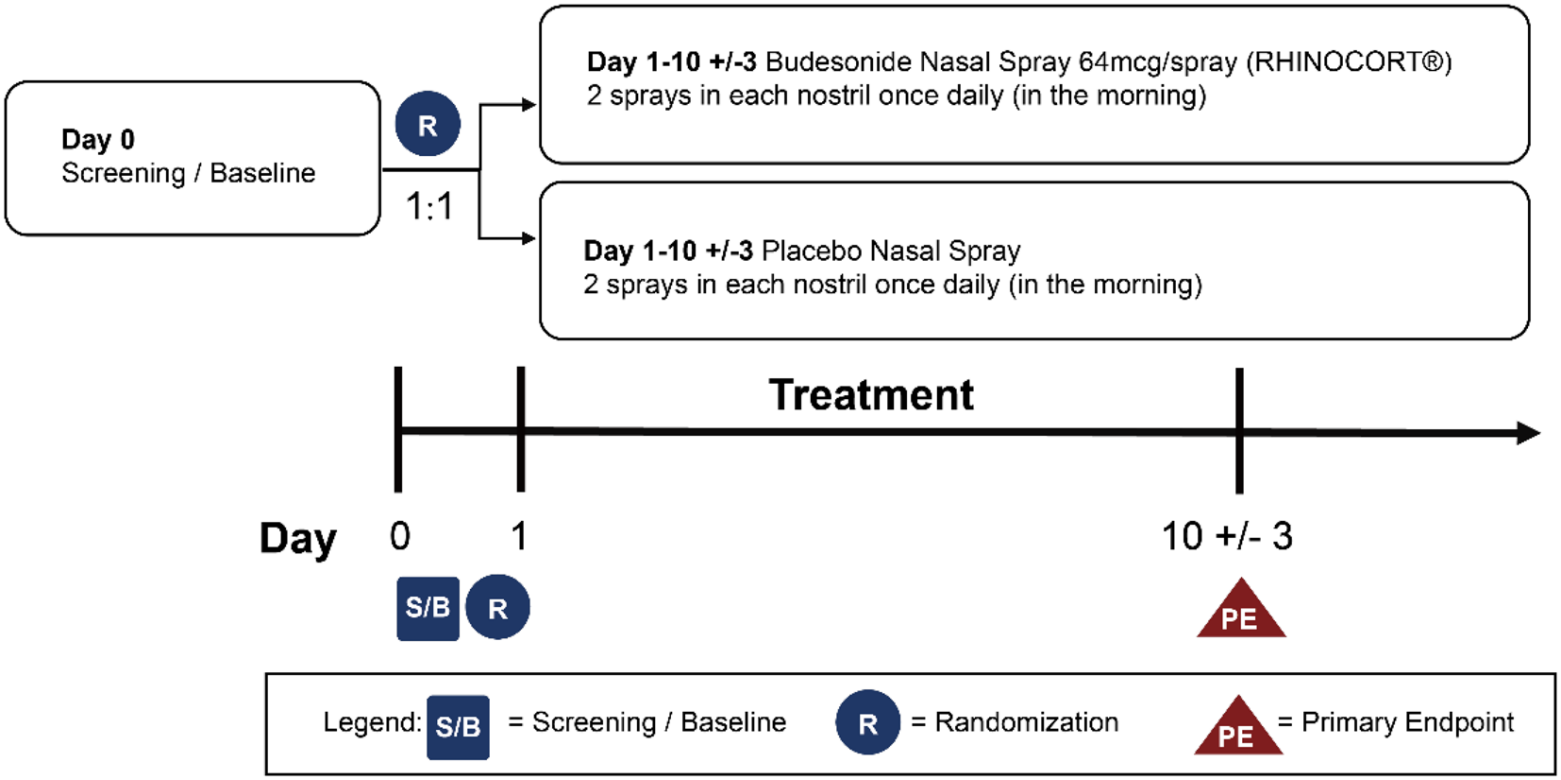
Schematic overview of the study

The subjects’ participation consisted of a screening phase (up to 2 days prior to Day 1), a double-blind treatment phase (from Day 1 to Day 10 [± 3 days]), and a post-treatment phase. This is to ensure that data was captured for this particular timeframe when the maximum effects of INS are expected*.* The subjects received budesonide 256 µg/day (two 64 µg/sprays in each nostril once daily) or placebo (two sprays in each nostril once daily), with the first dose administered on the morning following the baseline visit and continuing until the morning of the Final Efficacy Assessment (FEA). Each evening during the treatment phase, subjects’ 24-h reflective Total Nasal Symptom Score (rTNSS) [14] and outdoor exposure time were recorded at home in their e-diary (Fig. 1).

### Interim analysis

The planned total sample size was 230 subjects. Enrollment was interrupted by the COVID-19 pandemic on January 22, 2020, where 206 subjects (89.6% of the planned number) had been randomized. A protocol amendment was presented, with approval to add an interim statistical analysis. The purpose of the interim statistical analysis was to stop the study if the interim statistical results for the primary endpoint were sufficiently powered to demonstrate efficacy. The interim analysis, conducted by an independent third-party statistician and programmer, showed that the study could be terminated early since the primary efficacy endpoint demonstrated significant results. No subjects enrolled in the study had COVID-19, and the integrity of the study was not compromised.

### Efficacy and safety endpoints

The primary efficacy endpoint was the mean change from baseline in 24-h rTNSS averaged over the first 10 days of treatment. The major secondary endpoints were the subject-assessed global impression of change (SGIC) at the FEA, mean change from baseline in individual nasal symptom scores (nasal obstruction, secretion/runny nose, and itching/sneezing), mean change in 24-h reflective individual non-nasal symptom scores (cough, postnasal drip); and combined symptoms (nasal obstruction + itching/sneezing, secretion/runny nose + itching/sneezing, nasal obstruction + secretion/runny nose). The mean change from baseline Rhinoconjunctivitis Quality of Life Questionnaire (RQLQ) [15] at FEA; daily change in rTNSS for each day of the treatment period; physician-assessed global impression of change (PGIC) at FEA; and subject ratings of user experience and perception of treatment benefits were additional secondary endpoints. Safety was evaluated based on spontaneously reported adverse events (AEs) and routine physical and laboratory examinations.

### Airborne pollution exposure

The Air Quality Index (AQI) data were from the China National Environmental Monitoring Centre. Subject’s daily time outdoors was summarized by each day and treatment group. The daily average of each atmospheric factor and additional variables were listed by each day and each city.

### Statistical analysis

A sample size of 100 subjects per group was estimated to provide 90% power to detect an effect size of 0.47, which was estimated from previous studies [16]. This sample size also provided 82% power should the effect size be 0.41. Anticipating a 15% attrition, randomizing 230 subjects was planned.

The efficacy assessment was based on the Full Analysis Set (FAS), which includes all randomized subjects with baseline and at least one treatment diary data. The primary efficacy analysis for the change from baseline in 24-h rTNSS over the first 10 days treatment period was based on the mixed model for repeated measure (MMRM), including terms for treatment, day, center, and baseline rTNSS as covariates. The treatment difference and the 97.5% confidence interval (CI) for the treatment difference were estimated from the model. The same approach was used for analyzing individual nasal/non-nasal symptoms scores. To compare the efficacy results between the FAS and the Per-Protocol Set (PPS), an analysis of the PPS including only subjects with no major protocol deviations was also performed. An analysis of variance (ANOVA) including terms for treatment and center was used for Global Impression of Change. RQLQ was analyzed by ANOVA, including terms for treatment, center, and baseline as covariates.

**Fig. 2:**
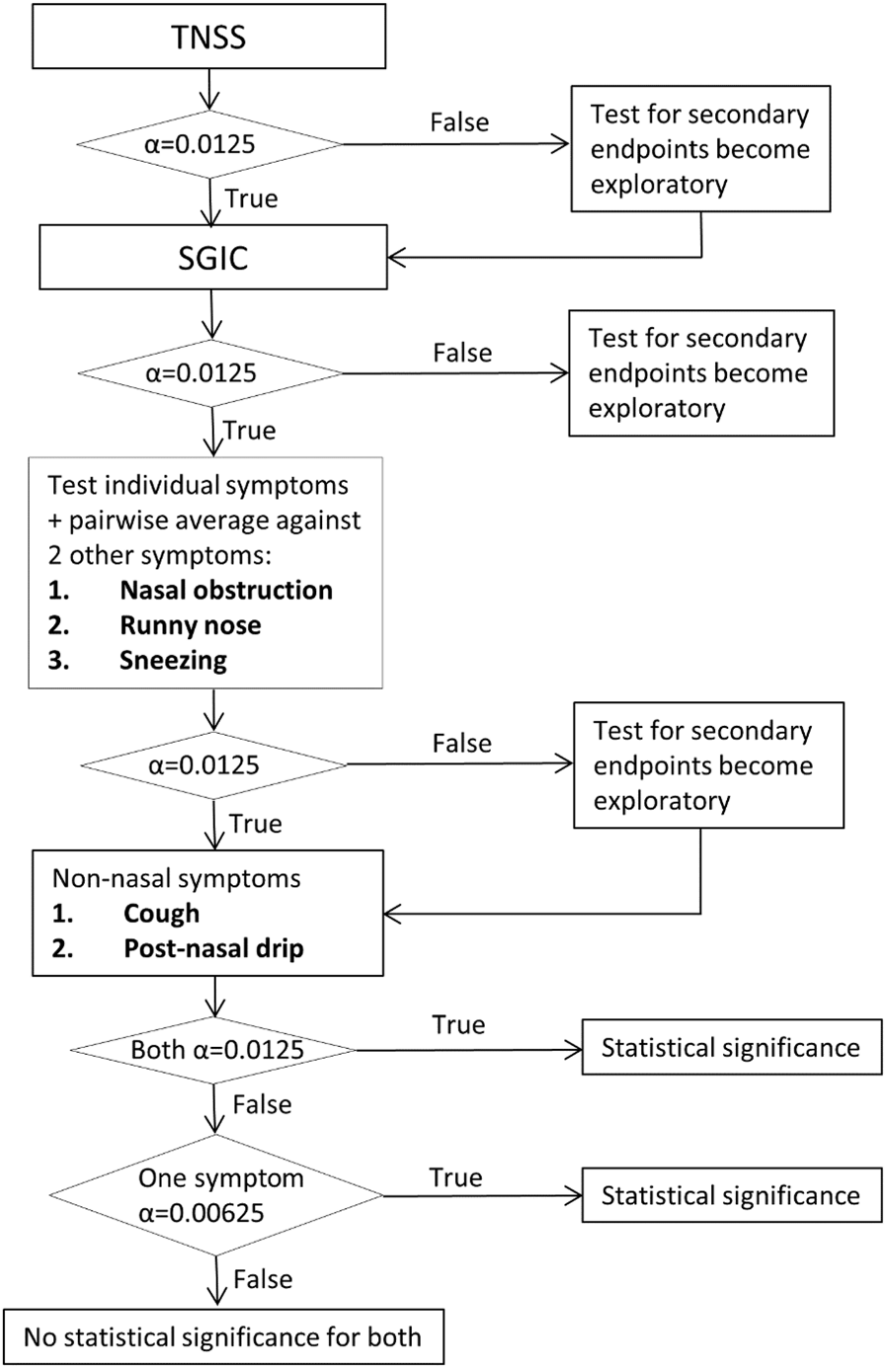
Sequential testing procedure to determine statistical significance for primary and secondary endpoints. *TNSS* total nasal symptom score, *SGIC* subject global impression of change. Alpha concentered are all one-sided, adjusted to half because of interim analysis

Due to the interim analysis, the alpha level was adjusted from 0.025 to 0.0125, one-sided. Sequential testing procedure was applied to rTNSS through individual nasal/non-nasal symptoms scores to control the overall type I error rate under 0.0125, one-sided. Individual nasal symptom scores were statistically evaluated using multiway averages, including pairwise averages with the other two nasal symptom scores (Fig. 2).

## Results

### Demographic and baseline characteristics

There were 206 treated subjects; 197 (95.6%) subjects completed the study. Six of the remaining nine subjects violated at least one inclusion criterion. Three subjects discontinued the study. There were no COVID-19-related protocol deviations. A study-level protocol deviation occurred wherein randomization was conducted centrally instead of being site-stratified. The randomization method and statistical analysis model remained valid. The estimation of the treatment mean differences remained unbiased but with a slight yet non-significant decrease in power (less than 2%).

The FAS included all 206 treated subjects randomly allocated to budesonide (N = 103) and placebo (N = 103). Demographic and baseline characteristics were generally balanced between the treatment groups (Table 1). All of the subjects were Chinese and non-smokers with no claims of tobacco exposure. Fifty-one percent (105/206) of the subjects were male. The mean age was 36.0 (range, 20 to 70 years). At screening, the mean 24-h rTNSS was 7.0. The mean value of individual nasal symptoms for nasal obstruction, secretion/runny nose, and itching/sneezing was 2.5, 2.0, and 2.5, respectively, in FAS subjects. No clinically relevant differences were observed in medical histories between treatment groups. For exploratory purposes, one study site performed nasal swabbing, revealing that more than half of the subjects at that study site were negative for eosinophils (budesonide: 16/26 [61.5%]; placebo: 11/21 [52.4%]).

**Table 1:** Demographic data and baseline characteristics

	Budesonide (N = 103)	Placebo (N = 103)
Age (years)	35.2 ± 10.03	36.9 ± 11.12
Sex (male)	50 (48.5%)	55 (53.4%)
BMI (kg/m^2^)	23.33 ± 3.47	23.96 ± 3.45
Outdoor exposure (hours)		
1–6	95 (92.2%)	92 (89.3%)
> 6, ≤ 8	5 (4.9%)	3 (2.9%)
> 8	3 (2.9%)	8 (7.8%)
Daily AQI exposure	110.2 (53.1 to 230.8)	111.6 (59.7 to 230.8)
Baseline rTNSS	6.8 ± 1.27	7.1 ± 1.04
Nasal symptom scores		
NO	2.5 ± 0.78	2.4 ± 0.72
Secretion/RN	2.4 ± 0.74	2.5 ± 0.67
Itching/SN	1.9 ± 0.80	2.1 ± 0.74
Non-nasal symptom scores		
Cough	0.88 ± 0.97	0.92 ± 0.93
Postnasal drip	1.61 ± 0.92	1.56 ± 1.00
Duration of treatment (days)	11.0 (3.0 to 12.0)	12.0 (6.0 to 12.0)
Allergy medical history	17 (16.5%)	13 (12.6%)
Hypertension	1 (1.0%)	1 (1.0%)
Diabetes	1 (1.0%)	0

*AQI* air quality index, *rTNSS* reflective total nasal symptom score, *NO* nasal obstruction, *SN* sneezing, *RN* runny nose

**Fig. 3:**
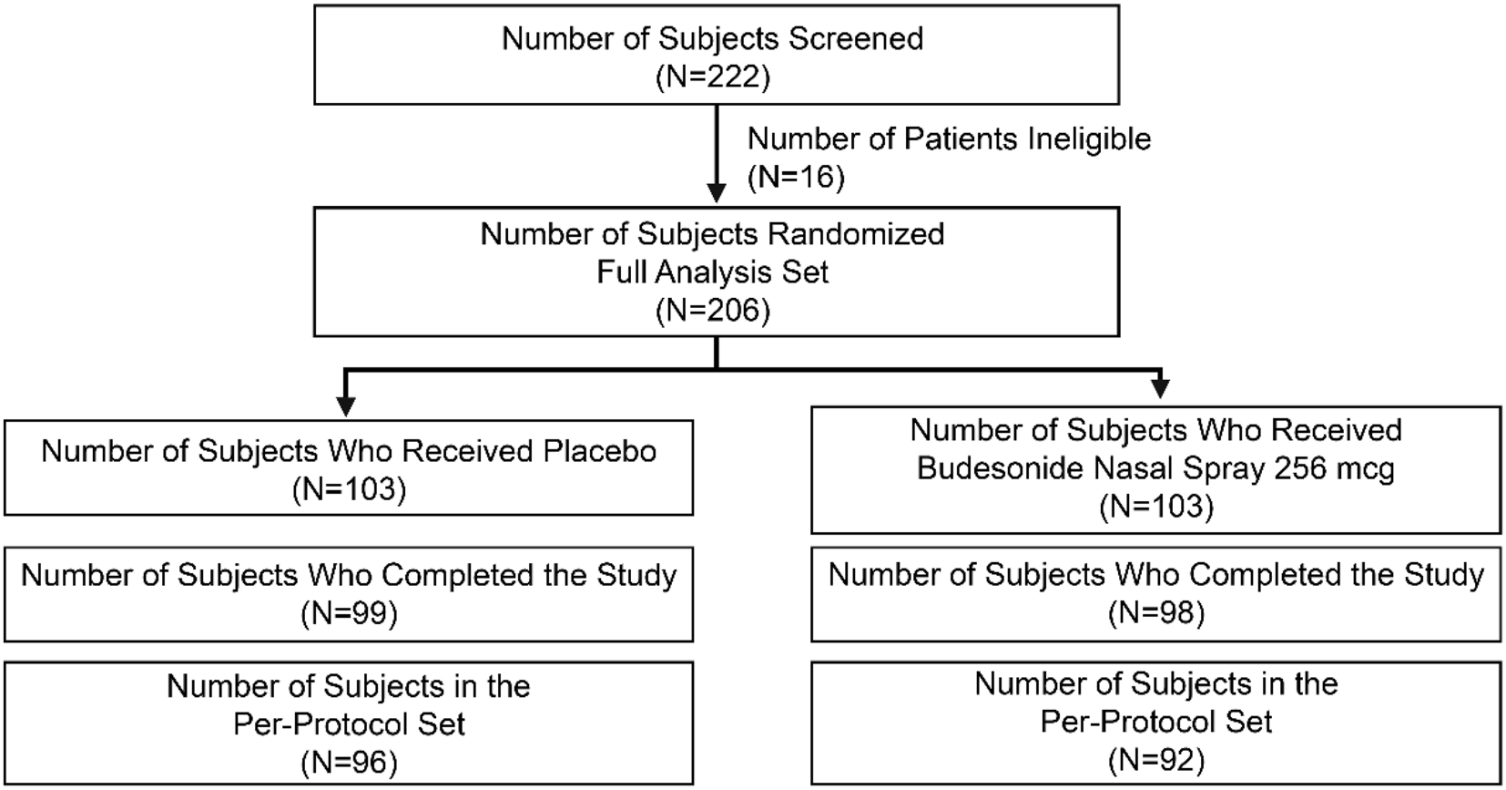
Subject disposition and analysis sets

A total of 16 subjects (7.8%; budesonide: 9 [8.7%]; placebo: 7 [6.8%]) had major protocol deviations and were excluded from the PPS. The PPS included 188 randomized subjects (Fig. 3).

The majority of subjects in both treatment groups spent 1–6 h outdoors each day. The average daily AQI exposure across all sites for each treatment group was 110.2 for the budesonide group (range: 53.1 to 230.8) and 111.6 for the placebo group (range: 59.7 to 230.8).

### Primary efficacy analysis of mean change from baseline in 24-h rTNSS

The median duration of treatment was 11.0 (range: 3.0 to 12.0) and 12.0 (range: 6.0 to 12.0) days for the budesonide and placebo groups, respectively. For the primary endpoint, budesonide produced a significantly greater reduction in rTNSS over the first 10-day treatment period compared with placebo (LS Mean: − 2.20 vs − 1.72, respectively; one-sided *P* = 0.0107; 97.5% CI 0.01, 0.94) (Table 2). A significant result, consistent with the FAS, was obtained by analysis based on the PPS (LS Mean: − 2.30 vs − 1.70, for budesonide vs placebo, respectively; *P* = 0.0031; 97.5% CI 0.11, 1.09).

**Table 2:** Primary and secondary endpoints (full analysis set)

	Budesonide (N = 103)	Placebo (N = 103)	*P*
	L.S. Mean (S.E.)^a^	L.S. Mean (S.E.)^a^	
rTNSS	− 2.20 (0.147)	− 1.72 (0.148)	**0.0107**
SGIC	− 2.35 (0.085)	− 2.20 (0.085)	0.1047
NO	− 0.73 (0.058)	− 0.60 (0.059)	0.0671
Secretion/RN	− 0.72 (0.054)	− 0.61 (0.055)	0.0781
Itching/SN	− 0.75 (0.055)	− 0.51 (0.056)	**0.0009**
NO + RN	− 1.45 (0.102)	− 1.21 (0.104)	0.0520
NO + SN	− 1.48 (0.102)	− 1.11 (0.103)	**0.0057**
RN + SN	− 1.47 (0.100)	− 1.12 (0.101)	**0.0065**
Cough	− 0.28 (0.052)	− 0.22 (0.053)	0.183
Postnasal drip	− 0.55 (0.051)	− 0.45 (0.052)	0.079

*rTNSS* reflective total nasal symptom score, *SGIC* subject-assessed Global Impression of Change, *NO* nasal obstruction, *SN* sneezing, *RN* runny nose

^a^Mixed model for repeated measure (MMRM) including terms for treatment, day, center, and baseline rTNSS as covariates, treatment-by-day, day-by-baseline interaction as well. The within-subject covariance is assumed as unstructured

Bold value indicate the *P* value < 0.0125 with statistical significance

### Secondary endpoint analysis of subject-assessed global impression of change (SGIC) at FEA

No significant difference was observed in the averaged SGIC between treatment groups (LS Mean: − 2.35 vs − 2.20, for budesonide vs placebo, respectively; *P* = 0.1047; 97.5% CI − 0.12, 0.41).

### Exploratory analyses

A pre-specified sequential testing procedure was utilized (Fig. 2). Because statistical significance was not demonstrated for SGIC, all tests for the following secondary endpoints were considered exploratory.

1. *Mean change from baseline in individual nasal symptom scores*
  Budesonide improved itching/sneezing averaged over the first 10-day treatment period (LS Mean: − 0.75 vs − 0.51 for budesonide vs placebo, respectively; *P* = 0.0009; 97.5% CI 0.07, 0.42). Budesonide also relieved the combined nasal symptom scores of nasal obstruction + itching/sneezing (LS Mean: − 1.48 vs − 1.11, for budesonide vs placebo, *P* = 0.0057, 97.5% CI 0.04, 0.69) and secretion/runny nose + itching/sneezing (LS Mean: − 1.47 vs − 1.12, for budesonide vs placebo, *P* = 0.0065, 97.5% CI 0.03, 0.67). The improvement in the remainder of the individual and combined nasal symptom scores did not reach statistical significance (Table 2).
2. *Mean change from baseline in 24-h reflective individual non-nasal symptoms*
  Budesonide did not significantly improve non-nasal symptoms compared with placebo (cough: *P* = 0.183 [97.5% CI − 0.10, 0.23]; postnasal drip: *P* = 0.079 [97.5% CI − 0.06, 0.26].

Other exploratory analyses pre-defined in the protocol are as follows.

**Fig. 4:**
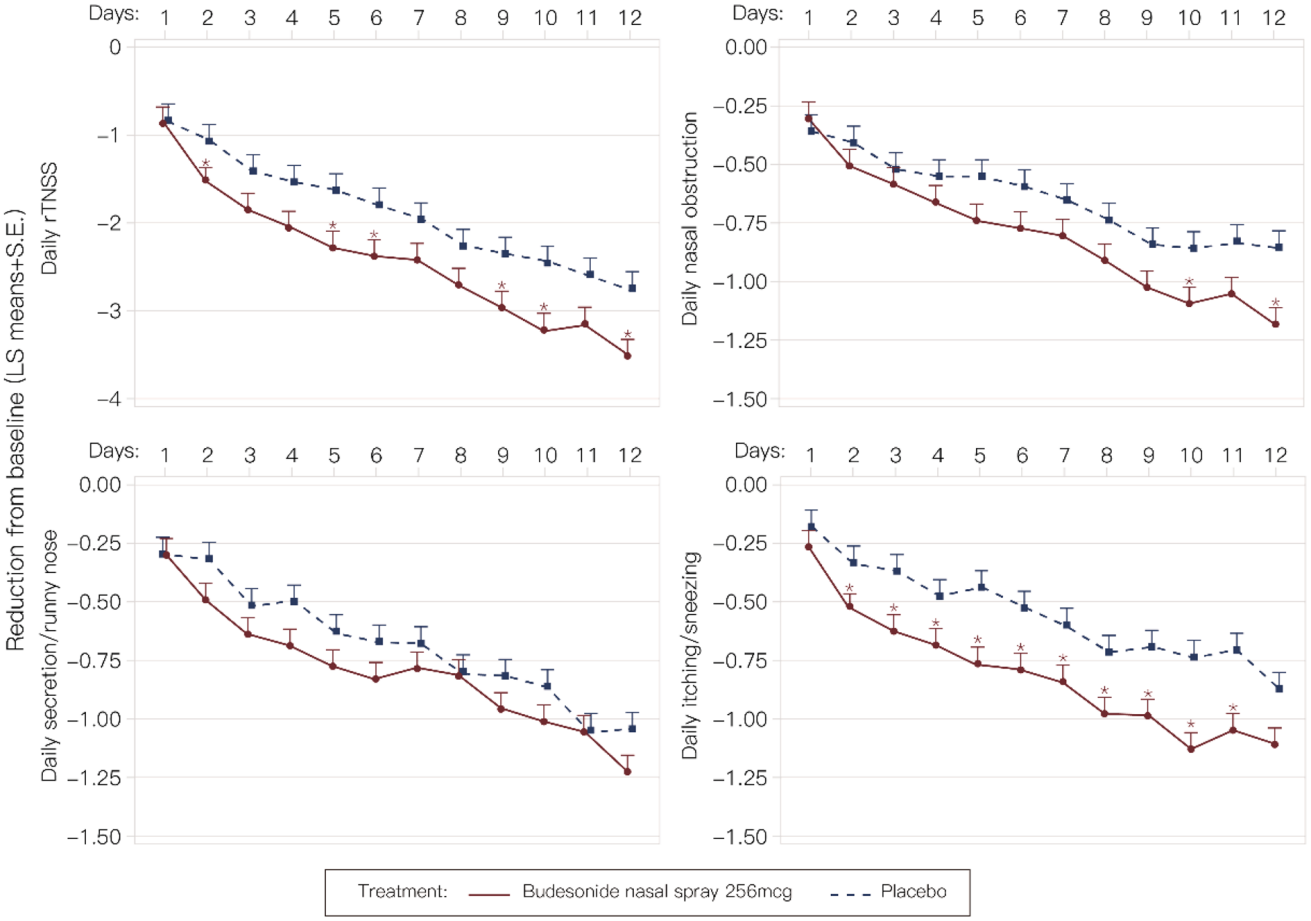
Daily change from baseline in reflective total nasal symptom score (rTNSS) and individual nasal symptoms (Full Analysis Set). **A** rTNSS. **B** Nasal obstruction. **C** Secretion/runny nose. **D** Itching/sneezing. Asterisks (*) denote statistical significance vs placebo. (*P* < 0.05, two-sided)

1. *Mean change from baseline for RQLQ at final efficacy assessment*
  At FEA, subjects’ RQLQ domain of eye symptoms were improved with budesonide vs placebo (LS Mean: 1.11 vs 0.87, respectively, *P* = 0.049, 95% CI 0.00, 0.48). Mean change from baseline for total RQLQ at FEA was 1.45 and 1.28 for the budesonide group and placebo groups, respectively (*P* = 0.142, 95% CI − 0.06, 0.39) (Table 3).
2. *Daily change in rTNSS*
  There was a greater daily rTNSS reduction from baseline for the budesonide group than that of the placebo group, achieving statistical significance on days 2, 5, 6, 9, 10, and 12 (*P* < 0.05, two-sided) (Fig. 4).
3. *Daily change in individual nasal symptoms*
  There appeared to be a general trend of greater daily reduction in individual nasal symptoms from baseline for the budesonide group compared to the placebo group (*P* < 0.05, two-sided) on days 10 and 12 for nasal obstruction and from days 2 through 11 for itching/sneezing. No significant difference between treatment groups was seen for secretion/runny nose (Fig. 4).
4. *Physician-assessed global impression of change at FEA*
  At FEA, a physician recorded PGIC for 205 subjects (budesonide: 102; placebo: 103). Of note, one subject treated with budesonide did not have an FEA visit. There was no significant difference in averaged PGIC between treatment groups (LS Mean: 2.41 vs 2.20, for budesonide vs placebo, respectively; *P* = 0.075; 95% CI − 0.02, 0.44). The highest percentage of subjects in both treatment groups had PGIC ratings of “minor control over symptoms” (budesonide: 40.8%; placebo: 37.9%) followed by “substantial control over symptoms” (budesonide: 30.1%; placebo: 34.0%). A greater proportion of subjects was reported by physicians as having “total control over symptoms” in the budesonide group (11.7% vs 1.9%, for budesonide vs placebo, respectively) and “no control over symptoms” in the placebo group (14.6% vs 26.2%, for budesonide vs placebo, respectively). None of the subjects in the budesonide group (0.0%) were physician-assessed as “symptoms were aggravated”; 1.9% of subjects in the placebo group were physician-assessed as “symptoms were aggravated.” (Additional file 1: Table S1).
5. *Subject ratings of user experience and perception of treatment benefits*

**Table 3:** Changes of the Rhinoconjunctivitis Quality of Life Questionnaire (RQLQ) scores at FEA from baseline

RQLQ (scores)^a^	Budesonide (N = 103)^b^	Placebo (N = 103)^c^	*P*
Total	1.45 (0.081)	1.28 (0.081)	0.142
Individual domains			
Activities	1.38 (0.093)	1.25 (0.093)	0.312
Sleep	1.65 (0.099)	1.46 (0.099)	0.181
Non-nose/eye symptoms	1.33 (0.086)	1.22 (0.086)	0.366
Practical problems	1.63 (0.113)	1.51 (0.113)	0.426
Nasal symptoms	1.67 (0.088)	1.47 (0.088)	0.109
Eye symptoms	1.11 (0.087)	0.87 (0.087)	**0.049**
Emotional	1.48 (0.099)	1.33 (0.099)	0.252

^a^Data presented as least square means (standard error)

^b^One subject did not finish the assessment, and one subject did not have an FEA visit

^c^One subject treated with placebo did not have baseline RQLQ scores

Bold value indicate the *P* value < 0.05 with statistical significance

A total of 204 subjects (budesonide: 102; placebo: 103) rated factors related to user experience and perception of treatment benefits at FEA. In the budesonide group, one subject did not finish the assessment, and one subject did not have an FEA visit. For the attribute of “able to breathe through the nose”, more patients in the budesonide group chose one of the top 3 choices over a seven-item scale, i.e., “strongly agree”, “agree”, or “somewhat agree”, compared with the placebo group (87.4% vs 77.7%), and a significantly higher rating was obtained in the budesonide group compared with the placebo group (5.44 vs 5.10; *P* = 0.018; 95% CI 0.06, 0.67). There were no significant differences for the other attributes (Additional file 1: Table S2).

### Safety

Thirteen subjects (budesonide: 5 [4.9%] subjects; placebo: 8 [7.8%] subjects) experienced at least one treatment-emergent adverse events (TEAE) during the study; a total of 14 TEAEs were reported (Table 4). There were no clinically relevant differences in the distribution of TEAEs across treatment groups. Three TEAEs were considered possibly, probably, or very likely related to the study drug (budesonide: 2; placebo: (1) All the reported TEAEs were mild, with no serious AEs reported.

**Table 4:** Treatment-emergent adverse events (TEAEs)

TEAEs, n (%)	Budesonide (N = 103)	Placebo (N = 103)
Subjects with ≥ 1 TEAE	5 (4.9%)	8 (7.8%)
Upper respiratory tract infection	3 (2.9%)	3 (2.9%)
Pharyngitis	1 (1.0%)	0
Epistaxis^a^	0	1 (1.0%)
Nasal congestion	0	1 (1.0%)
Nasal discomfort^a^	1 (1.0%)	0
Sneezing^a^	1 (1.0%)	0
Cupulolithiasis	0	1 (1.0%)
Headache	0	1 (1.0%)
Insomnia	0	1 (1.0%)

^a^Treatment-related AEs

## Discussion

This study aimed to assess the efficacy of intranasal budesonide in adults with self-reported rhinitis symptoms triggered/worsened by high airborne pollution. The results showed that budesonide resulted in a statistically significant higher reduction in 24-h rTNSS over the first 10 days of treatment compared with placebo. The results were consistent between the FAS and PPS, confirming the reliability of the result for the primary endpoint.

This is the first Phase IV study to provide direct evidence on the efficacy of an intranasal corticosteroid for rhinitis symptom relief during high airborne pollution. A prior 4-week, Phase IIa, randomized, double-blind, multicenter trial showed no significant difference in treatment effect between fluticasone furoate (FF) nasal spray 110 µg once daily, and placebo in patients with irritant (non-allergic) rhinitis triggered predominately by air pollution [17]. The authors analyzed that the lack of a treatment effect may be in part due to the overall good air quality present throughout the study or an insufficient dose or duration of FF. Unlike the smaller FF pilot study (n = 102), this multicenter, large-scale, budesonide study was sufficiently powered to test for a treatment difference between groups. In addition, the AQI in our study reached levels that were defined as unhealthy. Thus, our study adds to the evidence that budesonide significantly improves nasal symptom severity in subjects with rhinitis triggered or exacerbated by air pollution.

As outlined in the statistical analysis plan, power estimations were made only for the primary endpoint. Here budesonide produced a significantly greater reduction in rTNSS over the treatment period compared with placebo (LS Mean: − 2.20 vs − 1.72, respectively; *P* = 0.0107). Such relatively small but statistically significant difference between active and placebo therapies may be partly due to the placebo effect. Actually, previous studies on AR also showed a relatively large placebo effect and a less than 1.00 difference on nasal index score or rTNSS between active and placebo [16, 18]. Of course, it’s still better to conduct further studies with larger sample size population to confirm the conclusions here in the future.

Since statistical significance was not achieved in the SGIC as a secondary endpoint, subsequent endpoints became exploratory. Statistical significance is not consistent across these exploratory endpoints. Compared with placebo, budesonide significantly improved the exploratory endpoints of itching/sneezing averaged over 10 days (*P* = 0.001) as well as the combined scores of nasal obstruction + itching/sneezing (*P* = 0.006) and secretion/runny nose + itching/sneezing (*P* = 0.006). The improvement in the remainder of the individual and combined nasal symptom scores were not significantly different between the budesonide and placebo groups. There were also no significant differences in individual non-nasal symptoms between the budesonide and placebo groups. Taken together, these results may suggest that improvements in itching/sneezing are major contributors to the overall efficacy of budesonide during the 10-day treatment period. In addition, no significant difference was observed between treatment groups in the averaged change of nasal obstruction during the first 10 days, the statistical significance noted for congestion on Days 10 and 12 suggests that improvement in nasal obstruction may take a longer timeframe. A higher proportion of subjects in the budesonide group reported total control of symptoms (7.8% vs 3.9% for budesonide vs placebo, respectively), whereas a higher proportion of subjects in the placebo group reported no control over symptoms (9.7% vs 18.4%, budesonide vs placebo, respectively). These results were consistent with the PGIC results, but no significant difference in averaged SGIC or PGIC was observed between treatment groups.

Studies have shown that the changes in dispersion patterns and increased allergenicity of pollens and spores may be linked with climate change and air pollution. It poses a greater risk in sensitized people of developing allergic respiratory disease and aggravation of symptoms to those who are already affected [19, 20]. For patients with NAR, ambient physical or chemical stimuli alone irritate the nasal mucosa6. Even, rhinitis people without allergic sensitization are more likely to report more severe nasal symptoms when live in areas with higher levels of pollution compared with those with allergy [9] Nasal hyperreactivity is a common feature in both AR and NAR, generally regarded as nonspecific in NAR and phenotypic in AR [21, 22]. In present study, the participants were only asked to provide information about their previous allergy history (14.6%), confirmation of diagnosis was not performed primarily because the study is intended to target a real-world population of rhinitis sufferers from rhinitis symptoms triggered or worsened by airborne pollution regardless of the presence of underlying allergies. It is necessary to carry out in-depth research in a larger population with clear allergen information in order to specially analyze the impact of allergy on the conclusion of efficiency in the future.

Several explanations on the potential mechanisms of airborne pollution-induced rhinitis have been proposed. In-vitro culture of noninflammatory nasal mucosal tissue showed that exposure to PM2.5 might lead to loss of barrier function in human nasal epithelium through decreased expression of tight junction (TJ) proteins and increased release of proinflammatory cytokines [23]. The destructive effects of chronic airborne particulate matter exposure brought about by sinonasal airway barrier disruption and non-allergic eosinophilic inflammation have also been demonstrated in mice [24]. Even short-term haze exposure may lead to nasal inflammation and hypersensitivity, predominantly by Th2 cytokine-mediated immune responses, as demonstrated in a 5-day observational study involving healthy human volunteers [25]. One study was able to observe that concomitant exposure to environmental pollutants, such as diesel exhaust particles, and house dust mites may result to enhanced allergic airway inflammation characterized by increased airway eosinophilia, goblet cell metaplasia, accumulation of innate lymphoid cells (ILCs) and Th2 cells, type 2 cytokine production and airway hyperresponsiveness as compared to sole exposure with either of these two. This observed phenomenon appears to be relevant for this study as this was conducted during the winter season when pollution levels and predominance of perennial AR tend to be higher. Intranasal steroids are generally recommended as first-line treatment of rhinitis [26, 27] and have been proven to affect the restoration of the epithelial barrier function while resisting eosinophil-dominant inflammation. One study showed that early phase steroid-induced resolution involves inhibition of eosinophilia in total nasal tissue and lamina propria and CCL5-dependent recruitment of cells in the nasal mucosa in AR [28].

The decision to conduct this study in cities in northern China was based on historical air quality data. According to the 2019 Report on the State of the Ecology and Environment in China [29], the average proportion of days in the year that the northern Chinese cities of Beijing, Tianjin, Hebei, and their surrounding areas exceed the air quality standard (AQI ≤ 100) was 46.9% compared to that of the Yangtze River Delta at 23.5%. Additionally, air pollution is most serious and concentrated during the winter season, while seasonal allergen levels are very low. In this study, the average daily AQI exposure across all sites for each treatment group (110.2 for budesonide; 111.6 for placebo) suggests that the average air quality reached unhealthy levels for sensitive people [30, 31] during the study.

When the study enrollment was halted by the COVID-19 pandemic, the results of an interim analysis conducted by a third party demonstrated that the results were sufficiently powered to demonstrate efficacy for the primary endpoint. However, while early study cessation did not limit the results of this multicenter, randomized, placebo-controlled, double-blind study, it could have affected the turnout for the other secondary and exploratory efficacy measures. Another limitation is that the follow-up periods were short. It could limit the generalizability of the results since patients might require treatment for the entire pollution season, which lasts about 6 months.

Last but not least, although this study was carried out in Chinese population, to our knowledge, there is no evidence suggesting that the topical use of budesonide or other corticosteroids would have a different impact due to ethnicities. Moreover, pollution is a growing global problem, so the conclusions here are also likely to benefit patients with rhinitis caused by air pollution all over the world. Further studies are needed to investigate the effect of relevant atopic history and other comorbidities that may contribute to NAR as well as the local eosinophilic inflammatory status on the outcome of intranasal budesonide towards airborne pollution induced rhinitis.

## Conclusions

Overall, this study demonstrated the clinical effectiveness of intranasal budesonide 256 µg once daily in improving total nasal symptoms, and itching/sneezing in adults with rhinitis triggered or worsened by airborne pollution. Budesonide had an acceptable tolerability and safety profile, and no new safety concerns were identified.

## Supplementary Information

**Additional file 1:** **Table S1.** Physician-assessed global impression of change (PGIC) at FEA. **Table S2.** Subject ratings of user experience and perception of treatment benefits at FEA.

## Data Availability

To ensure patient confidentiality, the datasets generated and/or analyzed during the current study are not publicly available. The protocol, clinical study report, and datasets are available from the corresponding author on reasonable request.

## Abbreviations

AR: Allergic rhinitis
NAR: Non allergic rhinitis
INS: Intranasal steroid
FEA: Final efficacy assessment
rTNSS: Reflective total nasal symptom score
SGIC: Subject-assessed global impression of change
RQLQ: Rhinoconjunctivitis quality of life questionnaire
PGIC: Physician-assessed global impression of change
AE: Adverse event
AQI: Air quality index
FAS: Full analysis set
MMRM: Mixed model for repeated measure
CI: Confidence interval
ANOVA: Analysis of variance
TEAE: Treatment-emergent adverse event
