# Supplementary material for: Intranasal budesonide for rhinitis during a high airborne pollution period: a randomized controlled trial
Source: Allergy Asthma Clin Immunol. 2022 Jun 20;18:56. doi: 10.1186/s13223-022-00686-y (PMC9207822; doi:10.1186/s13223-022-00686-y)
Supplement: Supplementary file 1 — Additional file 1: Table S1. Physician-assessed global impression of change (PGIC) at FEA. Table S2. Subject ratings of user experience and perception of treatment benefits at FEA. [file 13223_2022_686_MOESM1_ESM.docx]

**Table S1.** Physician-assessed global impression of change (PGIC) at FEA

| **PGIC** | **Budesonide (N=102)*** | **Placebo (N=103)** | ***P*** |
| --- | --- | --- | --- |
| **Numerical Average^#^** | 2.41 (0.084) | 2.20 (0.085) | .075 |
| **Categorical, n (%)** |  |  |  |
| **Total control over symptoms** | 12 (11.7%) | 2 (1.9%) |  |
| **Substantial control over symptoms** | 31 (30.1%) | 35 (34.0%) |  |
| **Minor control over symptoms** | 42 (40.8%) | 39 (37.9%) |  |
| **No control over symptoms** | 15 (14.6%) | 27 (26.2%) |  |
| **Symptoms aggravated** | 2 (1.9%) | 0 (0.0%) |  |

*****One subject treated with budesonide did not have an FEA visit.

^#^ Numerical PGIC ranged from 0 to 4, with 0 representing symptoms aggravated and 4 representing total control over symptoms. Data presented as least square means (standard error).

**Table S2.** Subject ratings of user experience and perception of treatment benefits at FEA

| **Individual domains^#^** | **Budesonide**  **(N=103)*** | **Placebo**  **(N=103)** | ***P*** |
| --- | --- | --- | --- |
| **Able to breathe through nose** | 5.44±0.953 | 5.10±1.201 | **.018** |
| **Better night sleep** | 5.42±1.042 | 5.14±1.245 | .083 |
| **Confident with friends** | 5.34±1.013 | 5.17±1.133 | .215 |
| **Less distracted** | 5.39±1.049 | 5.22±1.171 | .289 |
| **Focus on things important to subjects** | 5.49±1.026 | 5.25±1.091 | .123 |
| **Confident at work** | 5.34±1.107 | 5.23±1.165 | .501 |
| **Help subject to stay focused at work** | 5.34±1.116 | 5.31±1.146 | .893 |
| **Daily responsibilities** | 5.33±1.031 | 5.30±1.037 | .831 |
| **Maintain appearance** | 5.51±1.006 | 5.27±1.182 | .128 |
| **Enjoy outdoors** | 5.50±0.955 | 5.31±1.112 | .236 |
| **Less bothered by environment** | 5.38±1.085 | 5.31±1.188 | .694 |
| **More comfortable outdoors** | 5.51±1.006 | 5.33±1.149 | .255 |

^#^Subject ratings ranged from 1 to 7 (1, strongly disagree; 2, disagree; 3, somewhat disagree; 4, neither agree nor disagree; 5, somewhat agree; 6, agree; 7. Strongly agree). Data presented as means ± standard deviations.
